# Management of decision of withholding and withdrawing life-sustaining treatments in French EDs

**DOI:** 10.1186/s13049-020-00744-7

**Published:** 2020-06-08

**Authors:** Marion Douplat, Laurie Fraticelli, Clement Claustre, Alexandra Peiretti, Patrice Serre, Magali Bischoff, Laurent Jacquin, Julie Freyssenge, Anne-Marie Schott, Karim Tazarourte, Soizic Frugier, Carlos E. L. Khoury, The LAT group, Maud Grezard, Jean-Damien Antoine, Odile Dumont, Elise Lhuillier, Luigi Pierro, Stephane Blain, Celine Prost, Piali Sen-Brachet, Achraf Khaldi

**Affiliations:** 1 Hospices Civils of Lyon, Emergency department, Lyon Sud Hospital, 165 chemin du Grand Revoyet, F-69495 Pierre Bénite, France; 2 UMR 7268 ADéS, Aix-Marseille Université / EFS / CNRS, Faculté de Médecine, 27 boulevard Jean Moulin, 13005 Marseille, France; 3 RESCUe-RESUVal Network, Lucien Hussel Hospital, Montée du Dr Chapuis, 38209 Vienne, BP 127 France; 4 EA4129, Systemic Health Pathway Laboratory, Lyon, France; 5 Emergency Department, Fleyriat Hospital, 01000 Bourg-en-Bresse, France; 6 Hospices Civils of Lyon, Emergency department, Edouard Herriot Hospital, 5 place d’Arsonval, F-69003 Lyon, France; 7 University Claude Bernard Lyon 1, HESPER EA 7425, Rhône, Lyon, France; 8 UMR 5600 Environnement Ville Société CNRS, University Jean Moulin Lyon 3, Rhône, Lyon, France; 9 Hospices Civils of Lyon, Pôle Information Médicale Evaluation Recherche, Lyon, France

**Keywords:** Decision making; emergency service, Palliative care, Health personnel

## Abstract

**Background:**

Decisions of withholding or withdrawing life sustaining-treatments in emergency department are part of current practice but the decision-making process remains poorly described in the literature.

**Study objective:**

We conducted a study in two phases, the first comprising a retrospective chart review study of patients dying in the ED and the second comprising survey study of health care workers at 10 urban emergency departments in France.

**Method:**

In a first step, we analyzed medical records based on fifteen criteria of the decision-making process grouped into four categories: the collegiality, the traceability, the management and the communication as recommended by the international guidelines. In a second step, we conducted an auto-administrated survey to assess how the staff members (medical, paramedical) feel with the decision-making process.

**Results:**

There were 273 deaths which occurred in the ED over the study period and we included 145 (53.1%) patients. The first-step analysis revealed that the traceability of the decision and the information given to patient or the relatives were the most reported points according to the recommendations. Three of the ten emergency departments had developed a written procedure. The collegial discussion and the traceability of the prognosis assessment were significantly increased in emergency department with a written procedure as well as management of pain, comfort care, and the communication with the patient or the relatives. In the second-step analysis, among the 735 staff members asked to take part in the survey, 287 (39.0%) answered. The medical and paramedical staff expressed difficult experience regarding the announcement and the communication with the patient and the relatives.

**Conclusion:**

The management of the decision to withhold or withdraw life-sustaining treatments must be improved in emergency departments according to the guidelines. A standard written procedure could be useful in clinical practice despite the lack of experienced difference between centers with and without procedures.

## Introduction

Deaths are often preceded by a decision of withholding or withdrawing life sustaining-treatments in emergency departments (ED) and mainly concerns patients over 80 years old with chronic underlying diseases, metastatic cancer or previous functional limitations [1–4]. Previous studies have shown that decisions of withholding and withdrawing life-sustaining treatments involved this patient profile in 80% of case [2, 3]. The decision making process to withhold or withdraw life-sustaining treatments has been examined at length within intensive care units (ICU) worldwide, but limited data exists in the ED setting [5–7].

Given the nature of the ED context, time management and chaotic work environment contribute greatly to the care a patient receives. Additionally, with limited data available concerning the patient’s state of health, information about previous functional limitations, and chronic diseases can be limited or absent entirely [8, 9]. Most of these patients are unable to communicate or practice autonomy and moreover there is a lack of advanced directives [10]. Ethics must be respected in this context, including but not limited to the principle of beneficence and non-malfeasance [11]. Thus, families are often asked to participate in the decisions about withdrawing or withholding life-sustaining treatments [2, 3]. There is a balance between medical and ethical consideration and also legal aspect which varies between countries. Guidelines have been established for the management of these decisions such as the involvement of relatives or the need for a collegial procedure, but there is limited data regarding the management of these decisions in the context of the ED [12–16] and the gap between real practice and guideline.

A previous study showed that physicians who were continually expected to determine the fate of patients receiving life-sustaining treatments reported a lack of professional emotional support in this process [17]. These situations could lead to emotional or psychological burnout and decreased job satisfaction [18]. Moreover, it has been demonstrated that communication and shared decision-making were key aspects relating to the transition from active treatment to end-of-life care [19].

For these reasons, we aimed to observe real practice in an ED French network, and the staff members feeling about decisions of withholding or withdrawing life-sustaining treatments.

We had a twofold goal:

- An evaluation of real practice about decisions of withholding or withdrawing life-sustaining treatments based on medical records,
- A survey administered to the staff members involved in these situations

## Methods

### Study design

We conducted a study in two phases, the first comprising a retrospective chart review study of patients dying in ED Network between July 2018 and December 2018 and the second comprising survey study of health care workers at 10 urban emergency departments in France. The institutional Ethic Committee approved the study and the study meets the STROBE statement [20].

### Study setting

**Fig. 1:**
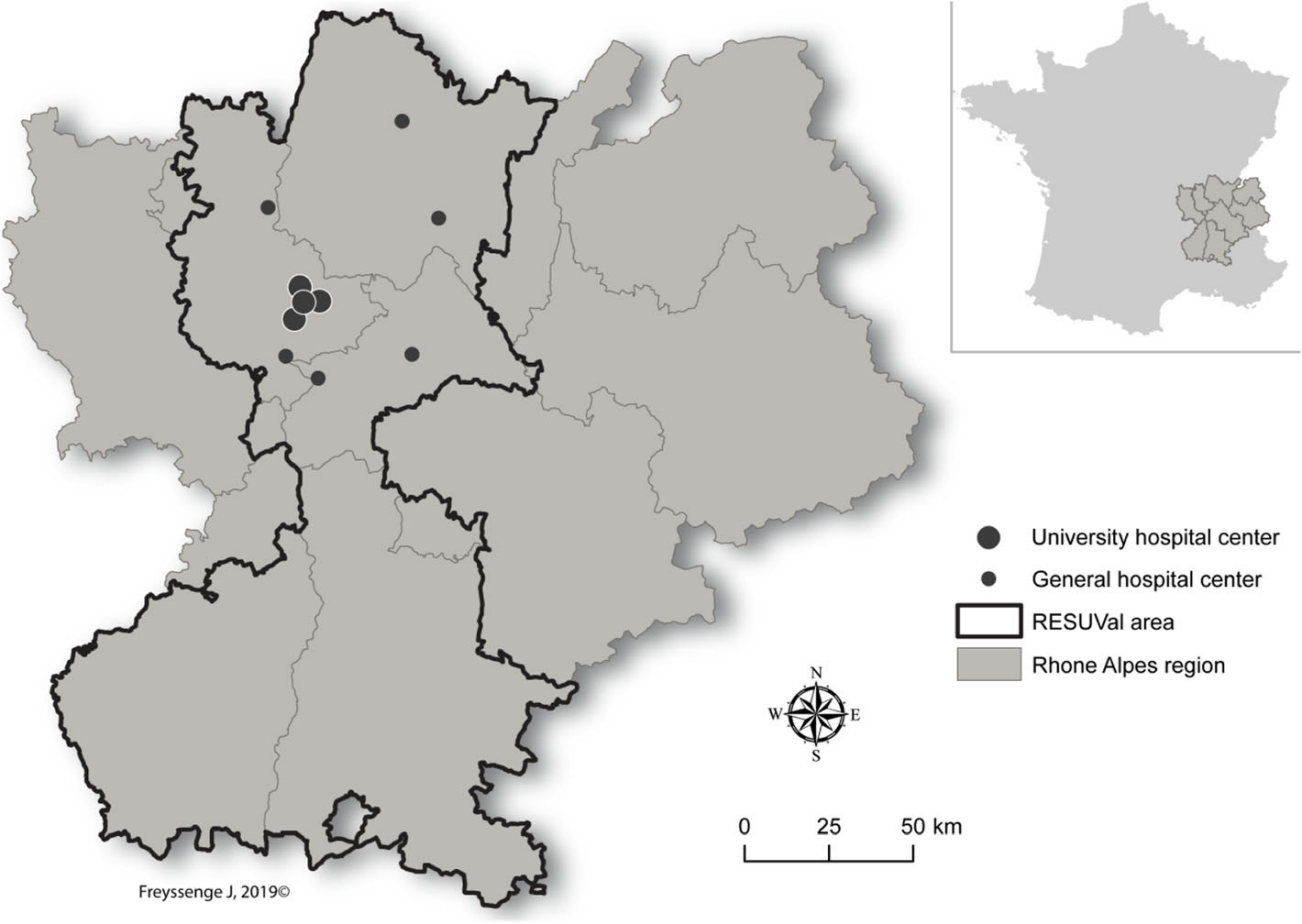
Geographic area of the RESUVal network

Funded by the French Regional Agency for health (ARS), the French emergency departments of the Rhone Valley in the Rhône-Alpes region (RESUVal) aimed to federate emergency physicians around guidelines to optimize quality of care and promote universal access. The RESUVal network covers a population of 3 million inhabitants with 38 ED spread over the territory. We randomly selected 25% of these ED for study participation. Therefore we enrolled 3 university and 7 general hospitals (Fig. 1).

### Data’s collection

#### Evaluation based on medical records

In each hospital, we included the medical records concerning adult patients who required a decision of withholding or withdrawing life-sustaining treatments in the ED between July and December 2018 and who died in ED or in emergency observation units.

In each center, a single physician independent of the study reviewed all the medical records of died patients in emergency department. He selected the records containing the following keywords: “withhold or withdraw life-sustaining treatments” or “limiting life-support therapies or palliative care”. These physicians were instructed at the beginning of the study by email for selection method. Withdrawal was defined as a discontinuation of treatments that had previously been implemented. Withholding was defined as a predetermined decision not to implement therapies that would otherwise be deemed necessary, because they were considered to be unable to modify the outcome in these particular instances.

Then, one physician independent of the study who was not involved in the selection of the medical records collected the data for all centers.

We analyzed the medical records based on fifteen criteria which have been proposed in in the context of intensive care units by the ethic section of the French society of intensive care (SRLF) [21] because none criteria were proposed in the EDs. The fifteen criteria are in agreement with the international guidelines and the ethical principles [13–17]. The definition of the validation for each criteria is detailed in the Table S1 in supplemental material.

**Fig. 2:**
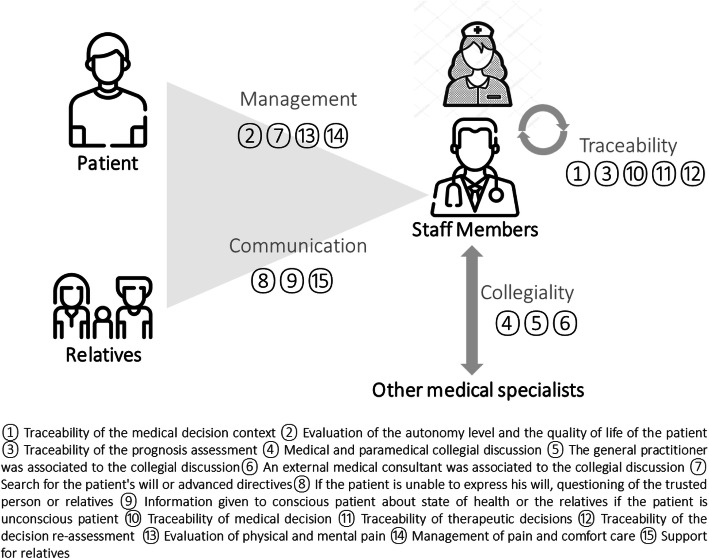
Fifteen criteria grouped in four categories for analyzing the decision-making process

We pooled the 15 criteria into four categories: the management, the collegiality, the traceability of the decision-making process and the communication between the patient, the relatives and the staff members (Fig. 2). The collegiality referred to whether non-physician, primary physician, and specialist physicians were documented as involved in the decision-making process. The involvement of an external medical consultant is required by the French law of February 2, 2016 on the new rights on the patient and the persons in end of life when the patient in unable to communicate. In the case of the external consultant usage -this is a service which is available depending on the type and size of the hospital and also on the day or night. It was available during the day for all 10 hospitals but not during the night. The traceability referred to documentation of the decision-making process. The management comprised evaluation of patients’ autonomy and patient’s care. The communication included documented the information given to the family and the support for relatives. We also noted if written procedure exist or not for each emergency department.

#### Survey administered to the staff members

We evaluated feeling, ethics concern and emotional burden trough a survey composed of twenty-one questions to the medical staff included physicians and residents, the paramedical staff included the nurses and the nurse’s aide. The listing had been retrieved by a medical and paramedical referent designated in each hospital. Data such as feelings and emotional responses were assessed with qualitative variables: not at all comfortable, rather uncomfortable, rather comfortable and completely comfortable. The survey was administered by email with a link to the e-questionnaire by the study coordination at the beginning of the study. An automatic email was sent every month during the study period to the medical and paramedical staff. The referent medical and paramedical had to promote the participation to the study in each ED. To ensure the anonymity, the answers were collected by the study coordination and not by the referent medical or paramedical. The details of the survey are in the additional material (S2).

### Statistical analysis

Baseline characteristics were described by frequencies and percentages for categorical variables, medians and interquartile ranges for continuous variables. Bivariate analysis was assessed using the Pearson Khi^2^ test for categorical variables and the Wilcoxon rank test for continuous variables. We also provided confidence 95% confidence range concerning the proportions observed of medical records from the 10 ED using Wilson’s method. Statistical analysis were performed using R 3.4.2 software (The R Foundation, Vienna, Austria). The threshold of significance was set at a *p*-value below 0.05.

## Results

### Real practice based on medical records

**Fig. 3:**
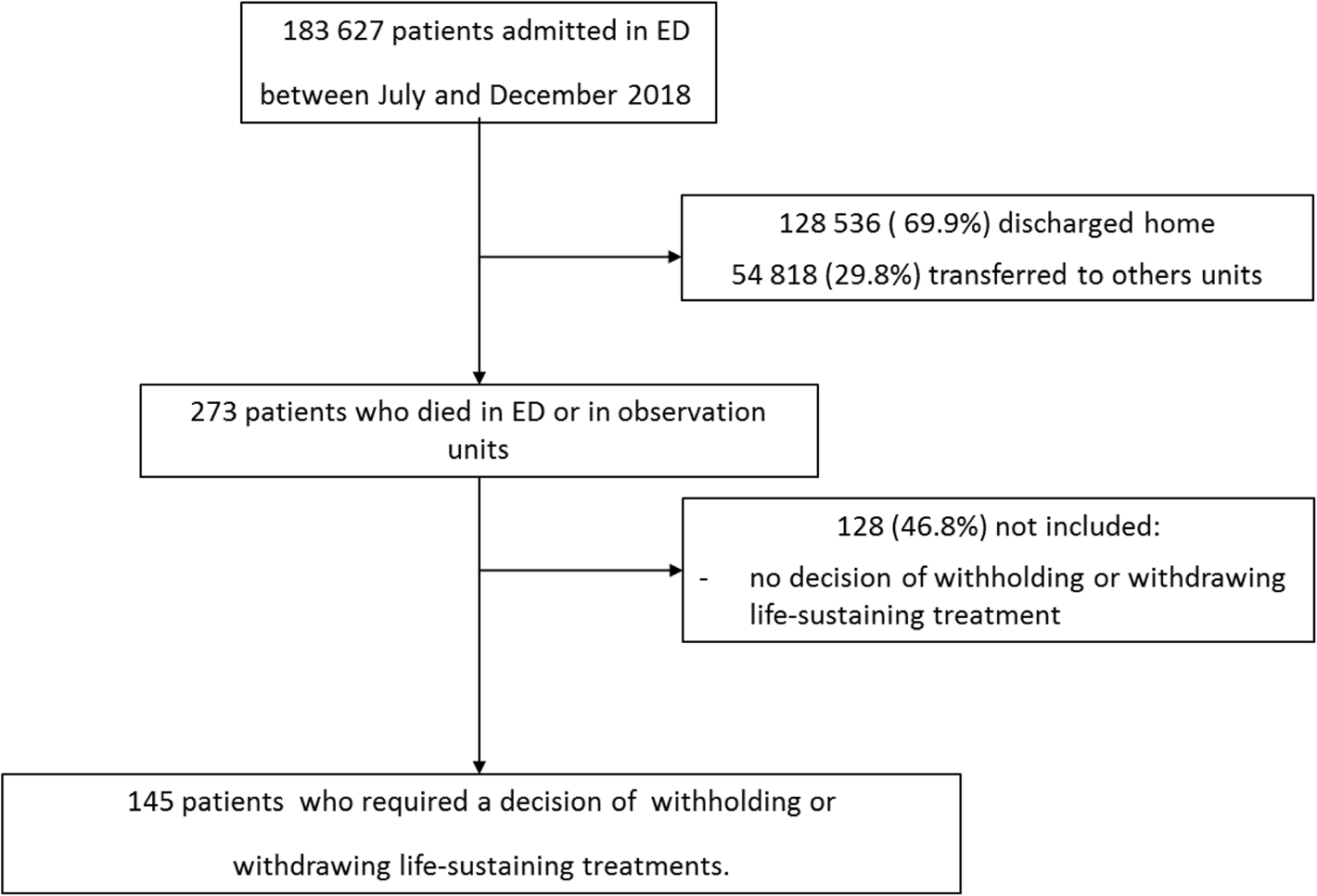
Trial profile of patients admitted to emergency departments during study period

Among the 183,627 patients who were admitted in ED during the study period, they were 273 deaths over the study period and we included 145 (53.1%) medical records of which 100 (68.9%) were from general hospitals. The flow chart is detailed on Fig. 3.

The traceability of the decision and the information given to conscious patient about state of health or the relatives if the patient is unconscious patient were the most reported points according to the recommendations whereas the involvements of the general practitioner and the support for relatives were rarely found. The decision-making modalities based on fifteen criteria are presented in Table 1.

**Table 1:** Decision-making modalities based on fifteen criteria in all centres

		*N* = 145 medical records	IC 95%
**Collegiality of the decision-making process**			
④	Medical and paramedical collegial discussion	60 (41.4%)	[33.7; 49.5]
⑥	An external medical consultant was associated to the collegial discussion	57 (39.3%)	[31.7; 47.4]
⑤	The general practitioner of the patient was associated to the collegial discussion	5 (3.4%)	[1.5; 7.8]
**Traceability**			
①	Traceability of the medical decision context	98 (67.6%)	[59.6; 74.7]
③	Traceability of the prognosis assessment	53 (36.6%)	[29.2; 44.6]
⑩	Traceability of medical decision	122 (84.1%)	[77.3; 89.2]
⑪	Traceability of therapeutic decisions after medical decision	29 (20.0%)	[14.3; 27.2]
⑫	Traceability of the decision reevaluation	34/50 (68.0%)	[54.2; 79.2]
**Management**			
②	Evaluation of the autonomy level and the quality of life of the patient	82 (56.6%)	[48.4; 64.3]
⑬	Evaluation of physical and mental pain	43/134 (32.1%)	[24.8; 40.4]
⑭	Management of pain and comfort care	78/117 (66.7%)	[57.7; 74.6]
**Communication**			
⑦	Search for the patient’s will or advanced directives	27 (18.6%)	[13.1; 25.7]
⑧	If the patient is unable to express his will, questioning of the trusted person, family or friends	54/136 (39.7%)	[31.9; 48.1]
⑨	Information given to conscious patient about state of health or the relatives if the patient is unconscious patient	111 (76.6%)	[69.0; 82.7]
⑮	Support for relatives	2 (1.4%)	[0.4; 4.9]

Three hospitals (1 university and 2 general hospitals) had a written procedure about the management of the decision and concerned 45 (31.0%) of the medical records. In ED with a written procedure, collegial discussion between the medical and paramedical staff (item 4) were more frequent (34 (75.6%) 95% CI [61.3–85.8] vs 26 (26.0%) CI [18.4–35.4]) as well as the involvement of an external medical consultant (item 6) (25 (55.6%) 95% CI [41.2–69.1] vs 32 (32.0%) 95% CI [23.7–41.7]).

The traceability of the prognosis assessment (item 3) was significantly better in ED with a written procedure (23 (51.1%) 95% CI [37.0–65.0] vs 30 (30.00%) 95% CI [21.9–39.6]). There was no difference between ED with and without procedure for the traceability of the context (item 1), the medical decision (item 10) and the decision re-assessment (item 12).

**Fig. 4:**
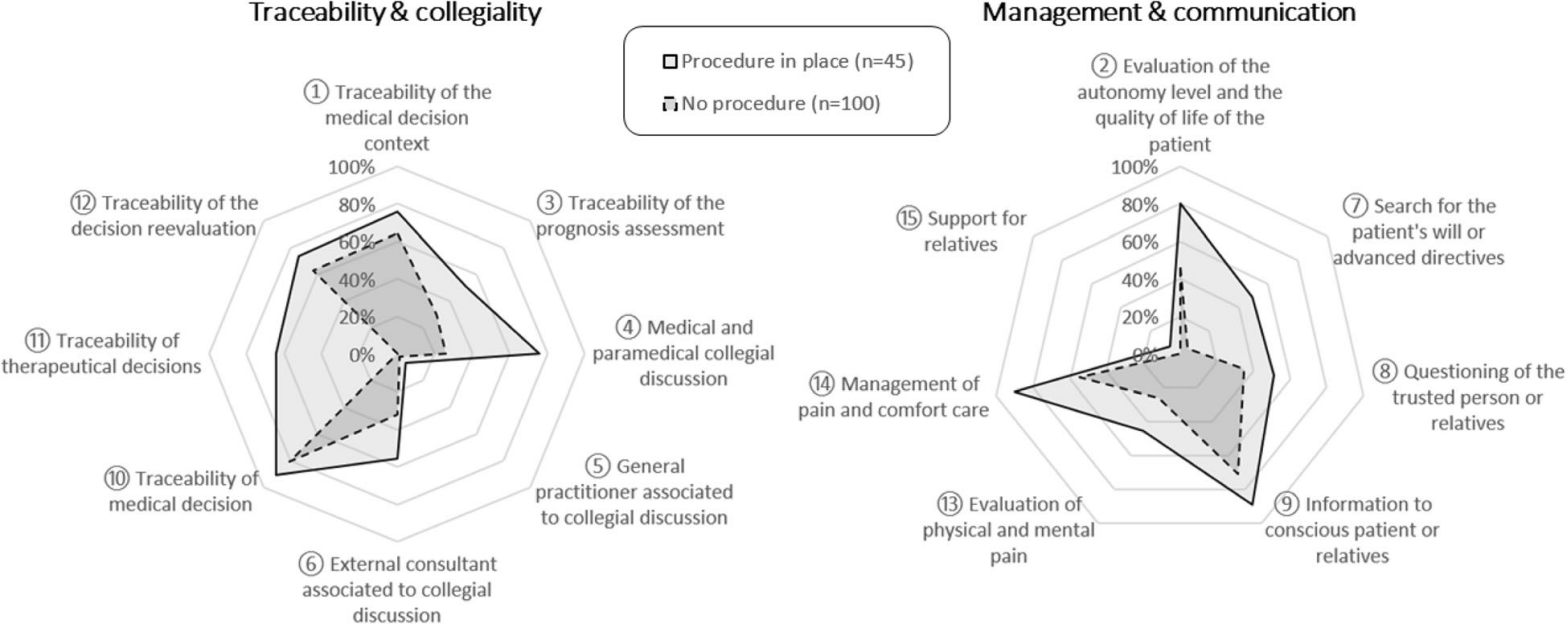
Decision-making modalities based on fifteen criteria according to the presence of a procedure or not

The evaluation of the level of autonomy (item 2) was better in department with a procedure; (36 (80.0%) 95% CI [66.2–89.1] vs 46 (46.0%) 95% CI [36.6–55.7]). In the same way, the evaluation of physical, mental pain (item 13) and the management of pain, comfort care (item 14) were performed for 19 (45.2%) 95% CI [31.2–60.1] and 36 (90.0%) 95% CI [76.9–96.0] respectively with a procedure vs 24 (26.1%) 95% CI [18.2–35.9] and 42 (54.55%) 95% CI [43.5–65.2] without. The information about state of health given to a conscious patient or to the relatives for unconscious patient (Item 9) was better with a procedure (40 (88.9%) 95% CI [76.5–95.2] vs 71 (71.0%) 95% CI [61.5–79.0]. The search of the advances directives (Item 7) was also improved with a procedure 22 (48.89%) 95% CI [41.2–69.1] vs 5 (5.00%) 95% CI [2.2–11.2] (Fig. 4).

### Survey to the staff members

Among the 735 staff members asked to take part in the survey, 287 (39.0%) answered. Respondent characteristics of the population are presented in Table 2. Among the medical staff, there were 91 physicians and 11 residents. Fifty-four (54.90%) of the doctors and 30.77% of the nurses work out-of-hospital at the MICU. It concerned in majority in our study, the same health team care who worked out-of-hospital and in the emergency department and cared the same patient. Thirty nine (13.6%) staff members received a training on this topic which was defined as at least 20 h over the last two years and mainly concerned the physicians for 30 (76.9%) of them. Seventy seven (27.4%) staff members reported that they were confronted with these decisions at least once a week and 102 (36.3%) once a month.

**Table 2:** Baseline characteristics of the survey population (*n* = 287)

	Medical (*n* = 102)	Nurse (*n* = 143)	Nurse ‘s aide (*n* = 42)
Demography			
Age in years, median [Q1; Q3]	36 [30.25; 43.5]	34 [28; 40]	40 [30; 47]
Male, n (%)	55 (53.92%)	31 (21.68%)	18 (42.86%)
Hospital Center			
University, n (%)	49 (48.04%)	58 (40.56%)	28 (66.67%)
General, n (%)	53 (51.96%)	85 (59.44%)	14 (33.33%)
Services			
Emergency department, n (%)	97 (95.10%)	140 (97.90%)	40 (95.24%)
Experience in years, median [Q1; Q3]	5 [2; 14]	5 [2; 10]	6.5 [4; 10.75]
Mobile Intensive Care Unit (MICU), n (%)	56 (54.90%)	44 (30.77%)	0 (0%)
Medical dispatch center, n (%)	22 (21.57%)	4 (2.80%)	1 (2.38%)
Intensive care unit, n (%)	4 (3.92%)	5 (3.50%)	3 (7.14%)
General medicine, n (%)	14 (13.73%)	5 (3.50%)	1 (2.38%)

The perception of the decision-making process according to the medical and paramedical staff is presented in Table 3.

**Table 3:** Perception of the management according to the medical and paramedical staff

	Medical (*n* = 102)	Paramedical (*n* = 143)	*p*-value
Systematic search for advance directives	59 (57.8%)	23 (16.1%)	< 0.0001
Systematic search for trusted person	51 (50.0%)	26 (18.2%)	< 0.0001
At least 2 physicians and 1 nurse in the decision making (including emergency physician)	59 (57.8%)	68 (47.6%)	0.1445
Place dedicated to the announcement	18 (17.7%)	24 (16.8%)	0.9961
Announcement > 15 min	23 (22.5%)	50 (35.0%)	0.0508

**Fig. 5:**
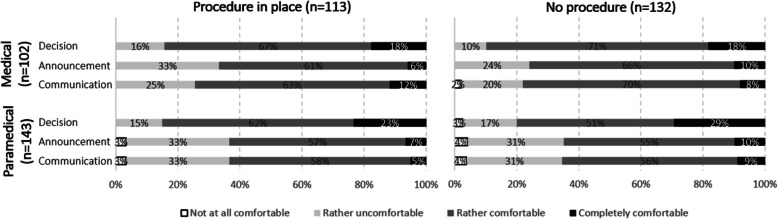
Experience of the management by the medical and paramedical staff according to the presence of a procedure or not

Regarding the feeling of the management by the medical and paramedical staff during the decision-making process, 158 (59.9%) 95% CI [53.8–65.6] expressed being rather comfortable, 64 (24.2%) 95% CI [19.4–29.8] completely comfortable and 39 (14.8%) 95% CI [11.0–19.6] rather uncomfortable. During the announcement, 148 (56.5%) 95% CI [50.4–62.4] were rather comfortable but 83 (31.7%) 95% CI [26.4–37.5] rather uncomfortable. When communicating with the relatives, 158 (58.1%) 95% CI [52.2–63.8] were rather comfortable and 81 (29.8%) 95% CI [24.7–35.5] rather uncomfortable. We found no significant difference with or without procedure for the feeling of medical and paramedical staff in the decision, the announcement and the communication (Fig. 5).

Among the staff members, 114 (39.7%) 95% CI [34.2–45.5] declared having no standard procedure. Among these members, 102 (89.5%) 95% CI [82.5–93.8] wished to have one in their services. Two hundred and thirty-seven (82.6%) 95% CI [78.2–87.1] staff members declared having no dedicated place for announcement and 140 (59.1%) 95% CI [52.7–65.1] wished to have one. Two hundred and seventy (94.1%) 95% CI [91.4–96.9] declared having dated knowledge, 213 (78.9%) 95% CI [73.6–83.3] of them wished for an update. Two hundred and fifty-nine (90.2%) 95% CI [86.3–93.2] declared an absence of debriefing sessions, 205 (79.2%) 95% CI [73.8–83.7] of them asked for a debriefing.

## Discussion

The management of the decision of withholding and withdrawing life-sustaining treatments in ED did not follow the recommendations of the guidelines and need to be improved. Our results strongly suggest there is a benefice of a procedure for improving the collegiality and the traceability. It enhances the communication between the staff members, the patient and the relatives, and the management of pain and comfort care. We also observed that a written procedure did not improve the experience of the staff members in these situations.

Few studies have focused on decisions to withhold or withdraw life-sustaining treatments but it appears in literature that it is a subject which concerns every ED in Europe and the USA [1, 8, 9, 22]. Moreover these decisions require taking into account the medical, ethical and legal aspect [23] and it is a challenge for the emergency staff. Our study is the first multicenter study to evaluate the management of these decisions with predefined criteria.

Our study has some limitations. Firstly, part one of the study is a retrospective study. It is possible that data could be missing from the medical records, especially concerning the collegiality of the decision-making process or the support for relatives. Some practices may be carried out but not written in medical records and underestimate the quality of practices. Moreover, as we didn’t planned an adjudication committee to select the medical records, it could have selection bias. However, the standardization of the selection method may partly have limited the inter-observer variability. Secondly, some of the 46.8% of ED deaths that did not document withholding or withdrawing care may have been futile cases and those discussions may have taken place but were not documented in a way that the chart review picked up on these cases. We found also a low ED mortality (0,15%) compared to USA (0.3%) but similar of another study in France (0.1–0.2%) [16] One explanation could be the specificity of France with the MICU which transfer the most critical patients directly from scene to ICU. Moreover, we didn’t included patients who had care withdrawn or withheld who did not die in the ED. Patients very well might have excellent end-of-life care, involving many of the 15 principles, and care may have been withheld or withdrawn, and the patient may have died out of the ED or emergency observation units. This point explain also the fact that we included 2–3 patients/months at each hospital which is similar with another study [3] but these results contrast with the survey answers. Staff members could be confronted with these decisions for patients who didn’t die in ED. These are good argument for the next study to have prospectively collected information.

Third, the criteria used to assess quality of care in the setting of withdrawal and withholding of treatment in French EDs have been published previously with legal standards for withdrawal and withholding of care in France which makes the practice environment there unique. One of the strengths of our retrospective study is that we do not observe the Hawthorne effect in which individuals modify an aspect of their behavior in response to their awareness of being observed [24].

Finally, we obtained a low response rate in the survey. In a precedent study on the same subject, we had a response rate of 59.4% from the physicians [17]. If we consider the response rate of the paramedical staff, it is lower than the physicians because the ratio nurse-nurse’s aide/physicians is 1.5. We can hypothesize that the paramedical team are uncomfortable on this topic and maybe the nurse’s aide feel less concerned. A qualitative study could be interesting to explore the barriers.

Our results strongly suggest a written procedure for assisting the collegial discussion between the medical and paramedical staff improves communication and quality of care and traceability. This is a key point considering the involvement of the nursing staff was insufficient during the decision-making process [2, 3, 17]. Despite international recommendations that have emphasized the need for collegial decision-taking and particularly the involvement of nurses this remains insufficient [12–17]. The poor implication of the GP in these decisions has been shown previously and more generally the lack of communication between GP and hospital [3, 25, 26]. The context of ED with the lack of time, the overcrowding and the absence of standardized ratio nurse/patients are obstacles to the decision-making process. Because of the legal aspects, these decisions required a good traceability to be in compliance with the law of each country and guidelines recommend that all discussions about end of life care decisions must be documented [12–16].

The improvement of the management of pain and comfort care with a procedure is also an important point for clinical practice. Previous studies have shown that palliative care are insufficient for patients who died in ED after a decision to withhold or withdraw life-support therapies and was administered to about half of them [27]. It has been demonstrated that an ED-based palliative care (PC provided directly in the ED) improve quality of life if palliative care was introduced early [28]. A qualitative study identified barriers to integrating palliative care in ED and showed the necessity to improve communication, as well as documentation about goals of care and symptom management [29].

Another primary point is the improvement of the communication between the teams and the patient and the relatives in our study. Both medical and paramedical face different challenges. Physicians have difficulty during the announcement whereas communicating with the family is more difficult for the paramedical staff. Moreover, physicians are more implicated the legal aspect than the paramedical. There is minimal literature concerning the end-of-life communication with relatives in ED [3]. In intensive care, it has been demonstrated that physicians lacked proper training for the skills required to communicate with patients, patient’s families and physician’s colleagues, including communication of futility [30]. Moreover, a poor communication between physicians and relatives could lead to complicated grief after death of a loved one in the intensive care units [30]. A brochure on bereavement and the use of a proactive communication strategy could lessen the effects of bereavement [31]. The need for adequate communication between family and staff members but also the need for communication training for teams has been demonstrated [32]. The debriefing piece could be an easy cost solution to improving how providers feel after taking care of patients at the end life.

Given the lack of consistency regarding life-sustaining practices, a procedural template which meets standardized international regulations (a checklist, for example), could be largely beneficial for staff members involved in the decision-making process. However some criteria in our study are not generable and specific for France like the involvement of an external medical consultant to the collegial discussion. This checklist should take into account the specificities of each country. A few studies have focused on the benefice of a written procedure in these situations. Sedillot et al. showed that a five-step protocol improved collaboration in the decision-making process and the transmission of information between staff and families [33].

While a written procedure can improve the decision-making process, some aspects remain difficult for the staff members. Indeed, the procedure alone made no difference in the survey results of the staff in their comfort level of both the management, making the announcement, and communicating with relatives. It is a compelling argument that written procedures are only part of the solution to improving end of life care in the ED, but that these procedures must also be combined with educational programming for the providers who are caring for these patients. Several ways for improvement are possible such as the skill communication training and further studies are needed to explore how to improve this point.

## Conclusion

The management of the decision of withholding and withdrawing life-sustaining treatment in emergency departments must be improved according to the international guidelines. A written and standardized procedure for ED could be helpful for homogenize clinical practice despite the lack of difference as experienced by the staff concerning the announcement and the communication with the relatives in these decisions. Communication training on the end-of-life issue could improve the experiences of the staff members as well as simulation-based training.

## Supplementary information

**Additional file 1: Table S1.** Evaluation criteria for the retrospective audit.

**Additional file 2.** Survey on the management of the decision of withholding and withdrawing life sustaining treatments in emergency departments.

## Data Availability

The datasets used and/or analysed during the current study are available from the corresponding author on reasonable request.
