# Supplementary material for: Management of decision of withholding and withdrawing life-sustaining treatments in French EDs
Source: Scand J Trauma Resusc Emerg Med. 2020 Jun 8;28:52. doi: 10.1186/s13049-020-00744-7 (PMC7282105; doi:10.1186/s13049-020-00744-7)
Supplement: Supplementary file 1 — Additional file 1: Table S1. Evaluation criteria for the retrospective audit. [file 13049_2020_744_MOESM1_ESM.docx]

Table S1: Evaluation criteria for the retrospective audit.

| Evaluation criteria | | Concerned actors | Validation of the criteria if: |
| --- | --- | --- | --- |
| 1 | Traceability of the medical decision context | Emergency physicians | The diagnosis must be precise and the stage of pathology evolution well established if the short-term vital prognosis is engaged because of an incurable pathology   - An intensification or continuation of treatment would be unreasonable or disproportionate in relation to the patient's situation: depending on the previous condition with in particular the autonomy evaluation, the probable sequelae taking into account the knowledge in the pathology and the reasoned discussion on the principle benefits/risks or even age. |
| 2 | Evaluation of the autonomy level and the quality of life of the patient | Patient  Family/relatives  Emergency physicians | - The patient’s medical records clearly mentioned the level of autonomy ( classified by physicians as complete, partial, or none if patients were bedridden) and the quality of life of the patient. |
| 3 | Traceability of the prognosis assessment | Patient  Family/ relatives  Emergency physicians | - The patient’s medical records clearly mentioned an evaluation of the prognosis from the actual health status, from the actual disease or comorbidities and prior therapeutic actions. |
| 4 | Medical and paramedical collegial discussion | Medical and paramedical actors | - The patient’s medical records clearly mentioned that the collegial discussion was realized during a dedicated time in the presence of the healthcare team concerned by the management of the patient. |
| 5 | The general practitioner of the patient was associated to the collegial discussion | Medical and paramedical actors | - The patient’s medical records clearly mentioned that the general practitioner was requested and participated to the collegial discussion, physically or by phone. |
| 6 | An external medical consultant was associated to the collegial discussion | Medical and paramedical actors | - The patient’s medical records clearly mentioned that a medical consultant, from outside the department, was requested to provide a written opinion about the care strategy. |
| 7 | Search for the patient's will or advanced directives | Patient  Family/ relatives  Emergency physicians | - The patient’s medical records clearly mentioned that the patient’s will has been searched if the patient is able to communicate - The patient’s medical records clearly mentioned that advanced directives has been searched if the patient is unable to communicate |
| 8 | If the patient is unable to express his will, questioning of the trusted person, family or friends | Family/relatives  Emergency physicians | - The patient’s will has been searched from the trusted person or from the family or relatives of the patient |
| 9 | Information given to conscious patient about state of health or the family/trusted person if the patient is unconscious patient or unable or under tutorship | Patient  Family/ relatives  Emergency physicians | - The patient’s medical records clearly mentioned that information in clear and appropriate terms has been provided to the patient, or to the trusted person, family or relatives, or if the guardian or judge of guardianship has been informed in the case of an unconscious or patient under tutorship |
| 10 | Traceability of medical decision | Emergency physicians | - The patient’s medical records clearly mentioned and dated the medical decision |
| 11 | Traceability of therapeutic decisions after medical decision | Emergency physicians | - The patient’s medical records clearly mentioned and dated the modalities of the decision (date and signature of the physician in charge). |
| 12 | Traceability of the decision re-assessment (LOS >24h) | Emergency physicians | - If the length of stay (LOS) is longer than 24 hours at the emergency department or to the short-term hospitalization unit, the patient’s medical records clearly mentioned that the medical decision is maintained or modified every 24 hours during the hospital stay |
| 13 | Evaluation of physical and mental pain | Emergency physicians | - The patient’s medical records clearly mentioned an evaluation of the physical and mental pain for the patient |
| 14 | Management of pain and comfort care | Emergency physicians | - The patient’s medical records clearly mentioned if an anxiolytic or analgesic has been prescribed and adapted |
| 15 | Support for relatives | Family/relatives  Emergency physicians | The patient’s medical records clearly mentioned at least three points:   - a dedicated room has been made available to family or relatives - a representative of the cult has been proposed - the access to the service has been open 24 hours a day - whether family or relatives have had appropriated interviews with the paramedical and medical team throughout the patient's progress |
