# Supplementary material for: Management of decision of withholding and withdrawing life-sustaining treatments in French EDs
Source: Scand J Trauma Resusc Emerg Med. 2020 Jun 8;28:52. doi: 10.1186/s13049-020-00744-7 (PMC7282105; doi:10.1186/s13049-020-00744-7)
Supplement: Supplementary file 2 — Additional file 2. Survey on the management of the decision of withholding and withdrawing life sustaining treatments in emergency departments. [file 13049_2020_744_MOESM2_ESM.docx]

**Survey on the management of the decision of withholding and withdrawing life sustaining treatments in emergency departments**

**This survey is to be answered anonymously by people working in an Emergency Department, confronted with** **decision of withholding and withdrawing life sustaining treatments**

| Yourself  Age : _________ Sex : 🞏 Woman 🞏 Man  Occupation : 🞏 Nurse 🞏 Nurse’s Aide 🞏 Physician 🞏 Resident  How long have you been working in an Emergency Department? : _______________ Years  Which position are you currently holding (several answers possible)? :  🞏 Emergency department 🞏 Mobile Intensive Care Unit  🞏 Medical dispatch center 🞏 General medicine  🞏 Intensive care unit 🞏 Other : _______________  Which hospital do you work at? ____________________ |
| --- |
|  |

| Completely comfortable  Rather Comfortable  Rather uncomfortable  Not at all comfortable  In general, how do you feel?   - during a decision of withholding and   withdrawing life-sustaining treatments 🞏 🞏 🞏 🞏   - during an announcement of withholding and withdrawing   life-sustaining treatments? 🞏 🞏 🞏 🞏   - while communicating with the family 🞏 🞏 🞏 🞏   In general, are modalities of withholding and withdrawing life-sustaining treatments ( hydratation, oxygenotherapy, antibiotherapy, blood test ) well defined ?  Often  Always  Sometimes  Never |
| --- |
| 🞏 🞏 🞏 🞏 |
|  |

| **In Practice**  **How often are you confronted with decisions of withholding and withdrawing life sustaining treatments?**  🞏Once a week 🞏 Several times a week 🞏 Once a month 🞏 Less than once a month  **Do you have a withholding and withdrawing life-sustaining treatments standard written procedure in your organization?**  🞏 Yes 🞏 No  **Have you already received a training (at least 20 hours over the last two years) concerning the management of withholding and withdrawing life-sustaining treatments in emergency departments?**  🞏 Yes : which one ___________________________________ 🞏 No | | |
| --- | --- | --- |
|  |  | |
| **Do you ask patients or their family for advance directives?**  🞏 Systematically 🞏 Often 🞏 Rarely 🞏 Never 🞏 I don’t know this concept | | |
| **Do you ask patients or their family whether a person of trust has been appointed?**  🞏 Systematically 🞏 Often 🞏 Rarely 🞏 Never 🞏 I don’t know this concept | | |
| **In your practice, what kinds of participants are generally involved in collegial reflexion about decision of withholding and withdrawing life sustaining treatments?**  🞏 Emergency physician  🞏 Intensive care physician  🞏 General practitioner  🞏 Nurse  🞏 Resident  🞏 Nursing aid  🞏Other : ______________________ | | **In your practice, what kinds of participants are generally involved in the announcement to the patient or the relatives?**  🞏 Emergency physician  🞏 Intensive care physician  🞏 General practitioner  🞏 Nurse  🞏 Resident  🞏 Nursing aid  🞏Other : ______________________ |
| **Where do announcement of withholding and withdrawing life-sustaining treatments occur ?(Several answers possible)**  🞏 Emergency box 🞏 Emergency corridor 🞏 Observation unit Room 🞏 Office 🞏 A dedicated place 🞏 Waiting room 🞏 Other  : ____________  **On average, how long does it take you to make the announcement?**  🞏 Less than 5 minutes 🞏 Between 5 et 15 min 🞏 Between 15 et 30 min  🞏 Between 30 min et 1 hour 🞏 More than one hour | | |
|  | | |

| **As of today, what are your needs about the management of the decision of withholding and withdrawing life-sustaining treatments in emergency departments?**  Yes No already set up   - A standard written procedure 🞏 🞏 🞏 - A dedicated place for the announcement 🞏 🞏 🞏 - An update of your knowledge 🞏 🞏 🞏 - Team debriefing sessions 🞏 🞏 🞏 |
| --- |
|  |

**Thank you for your answers**
